# Assessing the impact of tobacco control policies on smokeless tobacco uptake and use among secondary school students in South Asia: protocol for a feasibility study of conducting longitudinal surveys

**DOI:** 10.12688/f1000research.25796.1

**Published:** 2020-09-11

**Authors:** Masuma Pervin Mishu, Kamran Siddiqi, Ann McNeill, Mona Kanaan, Cath Jackson, Rumana Huque, Sushama Kanan, S.M. Abdullah, Fariza Fieroze, Suneela Garg, M. Meghachandra Singh, Amod L. Borle, Chetana Deshmukh, Zohaib Akhter, Laraib Mazhar, Zohaib Khan, Khalid Rehman, Safat Ullah, Lu Han, Anne Readshaw, Romaina Iqbal

**Affiliations:** 1 Department of Health Sciences, The University of York, Heslington, Yorkshire, YO10 5DD, UK; 2 National Addiction Centre, Institute of Psychiatry, Psychology & Neuroscience (IoPPN), King’s College London, London, SE5 8BB, UK; 3 ARK Foundation, Gulshan - 2, Dhaka, 1212, Bangladesh; 4 Department of Economics, University of Dhaka, Dhaka, 1000, Bangladesh; 5 Department of Community Medicine, Maulana Azad Medical College, New Delhi, 110002, India; 6 Departments of Community Health Sciences and Medicine, Aga Khan University, Karachi, 74800, Pakistan; 7 Institute of Public Health & Social Sciences, Khyber Medical University, Peshawar, Khyber Pakhtunkhwa, 25100, Pakistan

**Keywords:** Smokeless tobacco, secondary school students, adolescents, feasibility, longitudinal study

## Abstract

**Background:** Smokeless tobacco (ST) use is common among youth in South Asia where 85% of the world’s 300 million ST users live and use the most lethal ST forms. Little is known about the impact of tobacco control policies on the youth ST uptake in those countries. We planned to conduct longitudinal surveys among secondary school students to evaluate existing tobacco control policies on ST uptake and use, and a feasibility study for that prospective, observational cohort study.

**Study objectives: **(1) To demonstrate the feasibility of selection, recruitment and retention of schools and of study participants; (2) To assess the feasibility and acceptability of the study procedure and study tool (questionnaire); (3) To assess if the questionnaire can assess tobacco uptake and use, and the potential predictors of tobacco uptake and use of the envisaged main study.

**Methods and analysis:** The feasibility study will be conducted in two administrative areas within each of three South Asian countries: Bangladesh, India and Pakistan. We will use both quantitative and qualitative data collection methods. Eight eligible secondary schools will be randomly selected within purposively selected sub-districts from each country. We plan to conduct one baseline and one follow up survey among secondary school students, one year apart. At each time point, data on tobacco uptake and the potential predictors will be collected from students via self-administered questionnaires. The qualitative component will be embedded into the study with each round of data collection to assess the acceptability of the study instrument (questionnaire) and data collection methods, via focus group discussions with students and semi-structured interviews with schoolteachers.

Recruitment and retention rates, completeness of the questionnaires, frequencies and associations of tobacco use and explanatory variables will be reported. Data gathered from the focus group and interviews will be analysed using the framework approach.

## Introduction

Use of different types of tobacco products, both smoking and smokeless tobacco (ST), is a complex public health challenge for many countries ( United States National Cancer Institute, 2016). ST use poses complex problems, because its characteristics, patterns of use, health effects, production practices, and policy responses vary widely between countries and regions. In total, 85% of the world’s 300 million ST users live in South Asia and use the most lethal ST forms, which contain high levels of carcinogens, notably tobacco-specific nitrosamines ( Stanfill *et al.,* 2011). The use of these forms of ST leads to head and neck cancers and increases the risk of cardiovascular deaths ( Sinha *et al.,* 2016; Vidyasagaran *et al.,* 2016). Over 650,000 deaths per year, due to all causes, could be attributed to ST use worldwide; with 88% of this burden borne in South-Asia alone ( Sinha *et al.,* 2018). Despite the huge burden on health and the economy, ST remains largely neglected by policy makers and researchers, particularly in low and middle-income countries (LMICs). ST control has received less attention than smoking control and ST policies are poorly developed and have not been supported by high-quality research ( Siddiqi *et al.,* 2017). Compared to smoking, there is a huge policy implementation gap for ST ( Mehrotra *et al.,* 2019). The evidence for Framework Convention on Tobacco Control (FCTC) measures is mostly derived from cigarettes and the experiences in high-income countries. Little is known about their transferability to ST use in LMICs. Furthermore, most South Asian institutions do not have enough researchers or funds to carry out high-quality research in this area. Bangladesh, India, and Pakistan are three LMICs in South Asia where smoking and ST use have become an increasingly prevalent problem ( Islam *et al.,* 2014). Despite being signatories of the World Health Organisation (WHO) FCTC, these countries have made little progress towards tobacco control policies, in particular for ST ( Mamudu *et al.,* 2016; U.S. National Cancer Institute, 2016). For youth, the issue is even more complex as policies that work for the adult population might not be effective ( Crawford *et al.*, 2002). There is a need to develop a wider evidence-based response to FCTC for ST, particularly for youth in these countries.

A study assessing tobacco use among adolescents aged 12–15 years in 68 LMICs showed that mean prevalence of current tobacco use was 13.6%. About 10% of adolescents were cigarette smokers, while 8.1% were users of non-cigarette products that included ST ( Xi *et al.,* 2016). According to the recent Global Youth Tobacco Survey (GYTS), 4.5%, 9.0% and 5.3% of students were current ST users in Bangladesh ( GYTS Bangladesh, 2013), India ( GYTS India, 2009) and in Pakistan ( GYTS Pakistan, 2013), respectively. Since most adult tobacco users start ST use in adolescence, young people are targeted by the tobacco industry ( Wen *et al.,* 2005) and ST manufacturers ( Connolly, 1995; Tobacco Free Initiative & WHO, 2002). Thus, it is important to prevent the initiation of ST in adolescents, which would protect against the health risks of ST use in adult life. The WHO FCTC provides specific legislative measures to inhibit tobacco access and use by youth, increase awareness of the harm caused by tobacco and prevent the promotion of tobacco through sponsorship and advertisements ( WHO, 2003). Nonetheless, little is known about the impact of such tobacco policies on tobacco uptake and use among youth, due to lack of testing of effectiveness of policies in these countries. Within the current surveillance system, due to the cross-sectional design of the GYTS survey, it was only possible to look at the prevalence and associations but not a true evaluation of impact of the tobacco control policy. Moreover, questionnaires used in the GYTS ask specific questions for smoking but do not include similar questions on ST (sale, ST exposure outside the home and/or public places, health warnings on ST pack). Therefore, there is a gap of comprehensive assessment of ST.

We plan to conduct longitudinal surveys among secondary school students (year 6, 7 and 8 students), to test the impact of existing tobacco control policies. We will focus specifically on price and taxation policies, packaging and labelling policies for ST products, raising public awareness of tobacco-related harms, banning tobacco advertisement, promotion and sponsorship of tobacco, and policies banning tobacco sales to minors. The main aim of the study will be to test awareness of and exposure to policies and to assess their impact on ST use among adolescents over time compared to smoking. We have developed a comprehensive questionnaire that will cover both cigarette and ST use, and awareness and exposure to various tobacco control policies.

Feasibility studies are carried out before the main studies in order to test the processes involved (such as recruitment and retention of study participants and procedures for data collection) and estimate important parameters that are needed to design the main study ( Arain *et al.,* 2010). Most longitudinal studies have been carried out on smoking and very few of those included ST use. As very limited longitudinal studies have been conducted on high school students in Bangladesh, India and Pakistan to evaluate tobacco control policies particularly focusing on ST uptake, therefore, it is important to conduct a feasibility study before the envisaged longitudinal study.

## Study aim and objectives

### Aim

To assess the feasibility of conducting longitudinal surveys among secondary school students in Bangladesh, India and Pakistan to evaluate existing tobacco control policies on ST uptake and use among this group.

### Objectives

1. To demonstrate the feasibility of selection, recruitment and retention of schools and of study participants.

2. To assess the feasibility and acceptability of the study procedure and study tool (questionnaire).

3. To assess if the questionnaire can assess tobacco uptake and use, and the potential predictors of tobacco uptake and use to be assessed in the envisaged main study.

## Methods

We aimed to conduct a feasibility study of a longitudinal survey in secondary schools in three South Asian countries, Bangladesh, India and Pakistan, involving both quantitative and qualitative data collection. In this section the processes that have already been conducted are described in past tense, and those still to do are described in the future tense. The schools and students have been recruited and baseline data were collected between October 2019 and February 2020 and the data entry is still going on. We will revise the data collection tools in the light of feedback from the baseline data collection before carrying out follow-up data collection one year after the baseline data collection. We will revise the follow-up questionnaire if needed, based on the follow-up data collection experience.

### Sampling strategy

We used a multi-stage stratified random sampling strategy to recruit eight schools within each country. We purposively selected two administrative areas in each country, and from each administrative area, we selected one urban and one rural sub-district. From each selected sub-district, we selected schools that met the inclusion/exclusion criteria ( Table 1) and then stratified the schools by whether they were public or private and randomly selected one public and one private school from each sub district.

**Table 1.** Inclusion and exclusion criteria of selection of schools and students.

	Schools	Students
Inclusion criteria	• Follow mainstream curricula approved by the educational authorities. • Secondary schools that have year-six, seven, eight, nine and ten classes.	• Students of year 6-8 from the selected schools, who have the ability to give assent.
Exclusion criteria	• Have only primary school classes. • Teach in English medium only rather than national language. • Have already received training on a smoke-free intervention (or any other tobacco control intervention) from any previous project. • Religious or faith-based schools not following the prescribed curricula.	• Physical or mental disabilities • Learning difficulties and/or special learning- needs • Behavioral problems and/or conduct disorder • Serious medical condition which is either life-threatening or requires regular hospitalization

An invitation letter including brief information about the study was sent to the head teacher of each selected school taking part in the study. Interested schools were provided with a detailed information sheet and consent form. Those schools that provided written informed consent were recruited.

### Selecting the sample and recruitment

There are two groups of study participants: secondary school students and school staff – headteacher or a representative of the school and class teachers.

**Figure 1.**
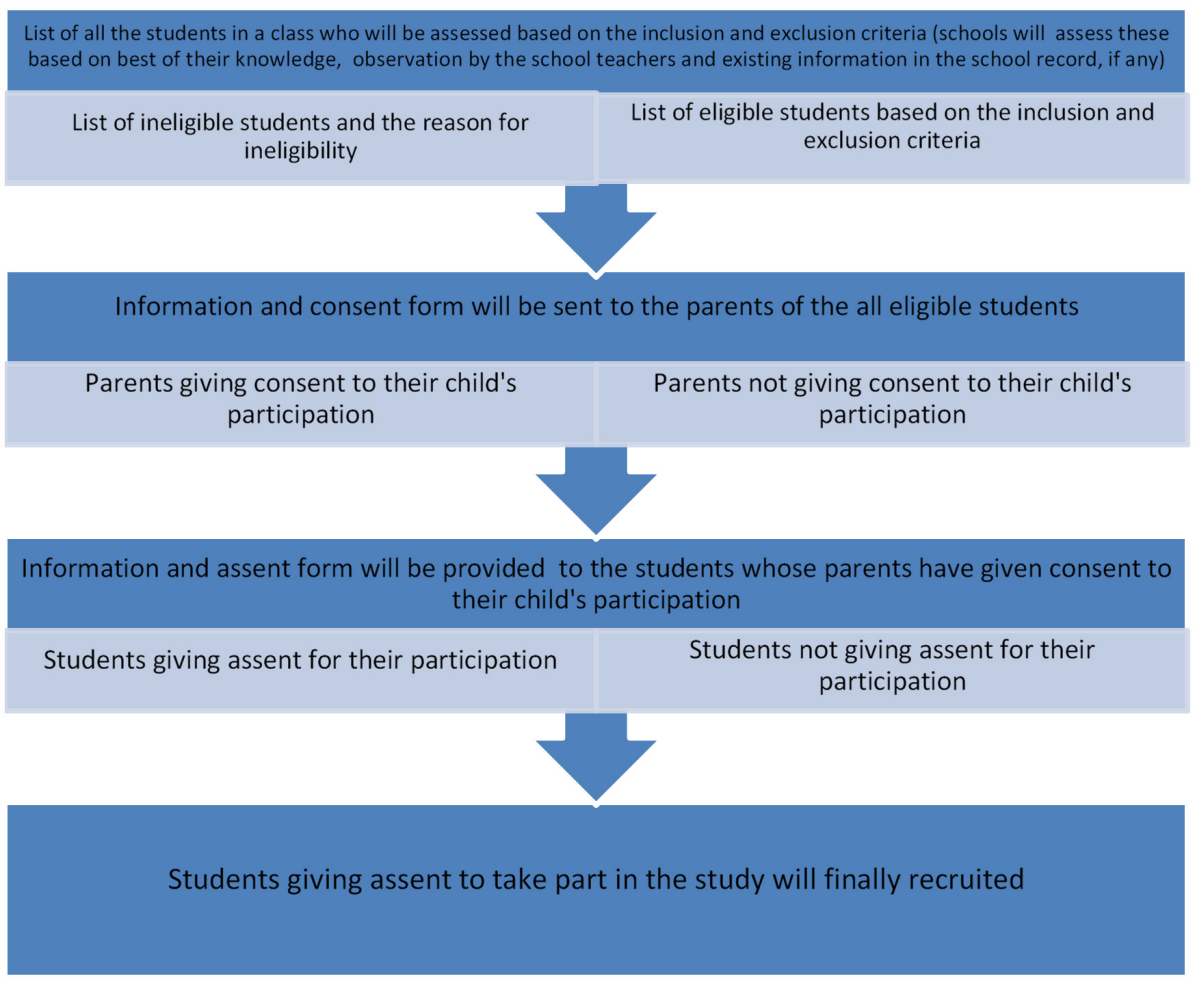
Stages of recruitment of the students.

In each selected school, three classes (class 6th, 7th and 8th) were selected. We aimed to recruit at least 25 students per class (at least 75 students from each school). As this was a feasibility study, we did not conduct a sample size calculation because we did not want to test any hypothesis. The steps taken to recruit eligible students are shown in Figure 1. First, we prepared a list of eligible students who met the inclusion criteria ( Table 1) and excluded those that fell into the exclusion criteria list. Once an eligibility list was prepared by the field investigators with the help of the class teachers, we gave the schools the required number of information packs containing an information sheet, and a parent/carer consent form to proceed with the recruitment. All students participating in this study were under 16 years old and therefore parental/carer consent was required for them to take part. The participating schools sent out the study information packs to the parents of all eligible students.

We asked parents/carers to discuss the study with their child and to indicate whether they were willing to let their child participate by sending back the signed consent form through the class teacher within one week. At the time of recruitment, children whose parents had provided consent were provided with an information sheet and an assent form so they could make an informed decision whether or not to participate. If students were unwilling, they could inform their class teacher. They were not coerced to consent. Students were asked to sign the assent form if they were willing to participate in the study. All participating students were given an enrolment number (including a code for school), which were recorded on the final list of eligible students and entered in the database.

For the qualitative student component, we used purposive and random sampling to select four schools (two urban, two rural). The intention was to conduct three focus group discussions (FGDs) per school, one per class (6th, 7th and 8th), with a mix of boys and girls. Students were randomly selected, having previously secured parental consent. Verbal assent was obtained from the selected students before the FGD commenced.

The headteacher or another nominated representative and the class teachers in all eight schools were approached to participate in a semi-structured interview. They were provided with an information sheet and asked to sign a consent form before the interview commenced. Where possible the class teachers were interviewed together to prompt discussion. 

### Outcomes to be measured

To address the objectives of the feasibility study, we will assess the following outcomes that have quantitative and qualitative components ( Table 2).

**Table 2.** Objective and outcomes.

Study objectives	Study outcomes (quantitative)	Study outcomes (qualitative)
Demonstrate the feasibility of selection and recruitment of schools and study participants	a. Time required to recruit schools and students. b. Recruitment rates for schools and students. c. Attrition rates at the various follow- up points for schools and students. d. Reasons for ineligibility of schools and students. e. Reasons for non-participation of schools and students.	
Demonstrate the feasibility and acceptability of study procedure and tool (questionnaire).	The rate of completed survey questionnaires.	Student, head teacher and class teachers’ feedback on feasibility and acceptability of the study procedure and tool (questionnaire).
Assess if the questionnaire is able to measure tobacco uptake and potential explanatory variables of the envisaged main study.	The proportion completing the questions on tobacco uptake and use and potential predictor of the envisaged main study.	The students’ feedback on their tobacco behaviour and perception.

***Main outcomes (related to objective 1)***

Quantitative:

a. Time required to recruit schools and students.

b. Recruitment rates for schools and students.

c. Attrition rates at the first follow-up point for schools and students.

d. Reasons for ineligibility of schools and students.

e. Reasons for non-participation of schools and students.

***Secondary outcomes (related to objective 2)***

Quantitative: The proportion of completed survey questionnaires.

Qualitative: Student, headteacher and class teacher feedback on feasibility and acceptability of the study procedure and tool (questionnaire).

***Secondary outcomes (related to objective 3).***

Quantitative: The proportion completing the questions on tobacco uptake and use and potential predictors of the envisaged main study, such as: level of knowledge and awareness on tobacco products and perceived tobacco use norms of the students, exposure to tobacco products, tobacco related health promotion, exposure to tobacco advertisements, perceived ease of access, affordability, and self-reported exposure to other peoples’ tobacco use. 

Qualitative: Student feedback on their tobacco behaviour and perception.

### Data collection methods

***Collection of data from the students.***

Quantitative data: A self-administered questionnaire for the students was developed and translated into local language and checked by a native speaker. The questionnaire included questions from the Global Youth Tobacco Survey (GYTS), Youth Tobacco Policy Survey (YTPS) and International Tobacco Control (ITC) survey questionnaire, and was pre-tested among 8–10 students per country before the baseline data collection. All data collection took place in the classroom. The investigator team visited the school and the pre-tested questionnaire was distributed among the students by the investigating team to all the eligible consenting students present in class. Those who were ineligible or did not give consent were moved to another classroom during data collection. The investigator team members helped the students with any further clarification. The privacy of students answering was ensured without others (teachers, classmates) overlooking their responses by giving prior instruction. The schoolteachers were not involved in questionnaire administration.

Qualitative data: After baseline data collection, the FGDs with students explored their views and experiences of being informed about the study, discussing the study with their parents, providing assent, and completing the questionnaire. After the follow-up data collection, the FGDs focused on students’ own and others’ tobacco uptake and use, influences on this. Topic guides were used to ensure consistency of discussion across schools, although the format was flexible to allow the students to raise additional issues they considered important. The discussions were conducted at the school in a private room by a field investigator and were digitally audio-recorded. Verbal assent was obtained from the selected students before their participation and recording.

***Collection of data from the headteacher or other school representative and class teachers.*** After baseline data collection, interviews with head teachers/other school representatives explored their views and experiences of hosting this study in the school. They also provided quantitative data at this time, on general information about the school, e.g. size, number of classes, the school tobacco policy and tobacco selling regulations. The interviews with class teachers focused on the process of informing parents and students about the study, organising consent, assent and survey administration. The field investigators conducted all interviews, which were digitally audio-recorded.

In addition, a logbook was maintained by each country throughout the process to record the time required to recruit schools and students, reasons for ineligibility of schools and students, and reasons for non-participation of schools and students.

### Data analysis

***Quantitative data.*** We will report recruitment, retention and attrition rates, percentage of completed questionnaires, missing data and summarised follow-up time. We will provide a diagram of flow of participants at baseline and at first follow-up. In addition, we will carry descriptive analyses for each phase of data collection. We will report the characteristics of students (e.g. demographic, socio-economic status, tobacco use) and information on ST uptake, and potential exposures. We will provide frequency and proportion for categorical variables and means and standard deviations for continuous variables. If a variable is skewed, we will provide medians and interquartile ranges and use graphical representation where appropriate. We will use STATA (2019) to carry the statistical analysis.

***Quantitative data.*** The interviews and FGDs were transcribed verbatim, and translated into English. A categorization matrix for each data set (head teachers/class teachers/students) was developed, organized by the steps of the study procedure. The data from the three countries has been coded into the same matrix, using Excel software ( Microsoft, 2018). The data analysis will be conducted using deductive content analysis ( Elo & Kyngäs, 2008).

## Ethical issues relating to the study and ethical approval received

In order to protect the study participants, the following provisions have been made/upheld:

### Recruitment

The most appropriate approaches to recruiting participants into the study were carefully considered. In addition, investigators involved in recruitment of study participants underwent suitable training and be provided with appropriate support. In order to ensure that participants of this study do not feel any inappropriate pressure or coercion, cautious attention was given to all recruitment procedures and materials.

### Consent

Consent forms and information sheets was carefully prepared and appropriate procedures was planned, in order to obtain a full-informed written consent in an acceptable and suitable manner. The participants acquired sufficient information and had the capacity to make the decision on whether to take part in the study. Furthermore, those participating in the study were informed of the right to stop their participation at any point throughout the study. Additionally, it was made very clear that participation, withdrawing from the study or not participating at all would not affect participants’ school results in any way.

### Risk, burdens and benefits

All research projects carry certain risks and burdens for the participants. Although this study does not involve any invasive procedures, it concentrates on tobacco use issues that potentially are of sensitive nature. Careful consideration was given in order to minimize the potential risks and burdens to participants. While developing the procedures and policies, every effort was made to reduce participants’ feeling of shame, guilt and pressure. Furthermore, attention was given to minimize participants’ time involvement. Additionally, the investigators participating in the study were appropriately trained and supported to decrease any burdens of taking part in the study. Consideration was given to avoid any pressure or coercion.

### Confidentiality

Every effort was undertaken to ensure confidentiality at all times throughout the study, including its design, conduct and reporting of the results. This study strictly followed ethical principles governing confidentiality. Participation in this study was anonymous so any name or any identifiable details would not be disclosed. The questionnaires were identifiable and were coded with a study enrolment number. The participants were assured that no names would be associated with the data, which would be kept in a locked secured facility.

Provision was made for indemnity by the investigator and sponsor.

The study obtained formal ethics review and approvals from the University of York (4-87/NBC-355/19/1695), the Indian Council of Medical Research (HMSC approval proposal ID 20182675, dated 13/04/2019', Bangladesh Medical Research Council (BMRC/ NREC/2016-2019/969, dated 07/01/2019.), National Bioethics Committee Pakistan (NBC: 4-87/NBC 355/Amend+Extension/20/1990?, dated 28/02/2019), and institutional level approval from Maulana Azad Medical College and associated hospitals, India; Aga Khan University, Karachi and, Khyber Medical University, Peshawar sites. Approvals from the participating school administrations have been obtained. 

## Data protection

Appropriate data protection and security procedures are put in place. Identifiable information collected on the consent form and codes were stored separately from the questionnaires. Interview and FGD data were entered using the IDs allocated to the schools and student participants.

All information collected during the course of the study was kept strictly confidential and will only be available to those involved in the research. Information was held securely on paper and electronically at the central research office. Any digital data was accessed only through use of security passwords. The researchers also complied with all aspects of related Data Protection Acts.

## Plans for dissemination of the findings once completed

We will disseminate the findings to academic audiences via publication in open access, high impact, and peer-reviewed scientific journals of relevant discipline and via related scientific presentations at national and international conferences and seminars. We will also disseminate to non-academic audiences, like national and regional stakeholders for tobacco control in SEARO and EMRO regions, community representatives and local administrations and participating schools and families.

## Data availability

### Underlying data

No data are associated with this article.
