# Supplementary material for: Protocol for a feasibility study of longitudinal surveys to assess the impact of policies on tobacco use among school-going adolescents in South Asia
Source: F1000Res. 2022 Mar 1;9:1123. Originally published 2020 Sep 11. [Version 2] doi: 10.12688/f1000research.25796.2 (PMC9274015; doi:10.12688/f1000research.25796.2)
Supplement: Peer review history

# Peer review: Reviewer response for version 2

**DOI:** 10.5256/f1000research.121646.r125841

**Published:** 2022-07-11

**Authors:** Wahyu Septiono

**Affiliations:** 1 Faculty of Public Health, Universitas Indonesia, Jakarta, Indonesia

No further comment.

Is the study design appropriate for the research question?

Yes

Is the rationale for, and objectives of, the study clearly described?

Partly

Are sufficient details of the methods provided to allow replication by others?

Yes

Are the datasets clearly presented in a useable and accessible format?

Not applicable

Reviewer Expertise:

tobacco control, health policy, statistics, epidemiology

I confirm that I have read this submission and believe that I have an appropriate level of expertise to confirm that it is of an acceptable scientific standard.

# Peer review: Reviewer response for version 2

**DOI:** 10.5256/f1000research.121646.r125840

**Published:** 2022-03-10

**Authors:** Praveen Jodalli

**Affiliations:** 1 Department of Public Health Dentistry, Yenepoya Dental College, Mangalore, Karnataka, India

Approved and accepted.

 All the comments done are addressed by the author.

Is the study design appropriate for the research question?

Yes

Is the rationale for, and objectives of, the study clearly described?

Partly

Are sufficient details of the methods provided to allow replication by others?

Yes

Are the datasets clearly presented in a useable and accessible format?

Partly

Reviewer Expertise:

Tobacco control, surveillance, public health, dental public health, clinical dentistry

I confirm that I have read this submission and believe that I have an appropriate level of expertise to confirm that it is of an acceptable scientific standard.

# Peer review: Reviewer response for version 1

**DOI:** 10.5256/f1000research.28468.r84185

**Published:** 2021-05-18

**Authors:** Wahyu Septiono

**Affiliations:** 1 Faculty of Public Health, Universitas Indonesia, Jakarta, Indonesia

Comments:

1. "Thus, it is important to prevent the initiation of ST in adolescents, which would protect against the health risks of ST use in adult life." This statement is not quite strong as authors only wrote about the ST prevalence which are 4.5% - 9.0% and added a reference about early smoking in adolescence. Perhaps authors can incorporate the prevalence over years in order to make this statement stronger and ST is a very important issue.
2. I am not really familiar with the educational system/stage in the study area. Is it similar in those three countries? Please add more detail about this.
3. For the FGD, please be more specific. How many participants per FGD?
4. In order to increase the validity (I may say the honest response) authors have clarified that schoolteachers were not involved in questionnaire. However, I think there is a missing important point here. Were teachers also in the class or around students during the data collection? Teacher presence may give less comfort ability for students to fill in the questionnaire and this leads to weak validity. Authors may consider this.
5. I would prefer to mention which version of STATA that was used.
6. Longitudinal study is a quite long study and this study has mentioned that the follow up will be undertaken the next year (one year). I wonder if students move to another school due to higher educational stage, will there be any follow ups or will they be declared missing? The investigators need to consider this.
7. Although this is a feasibility study, I wonder whether the instrument for the quantitative study has been piloted or not. If it was not piloted, I may say the study outcomes are incomplete. Translating questionnaire into a local language especially for a study among adolescents is a bit challenging. Authors need to add more detail about this (e.g., piloting the translation, third party who translated, etc). I think this should be included as the study outcome. For example, 1) during the survey how many students were asking specific questions because some words or sentences from the local language in the questionnaire that they might not understand, and 2) feedbacks or comments from students about each specific questions during the FGD.

Is the study design appropriate for the research question?

Yes

Is the rationale for, and objectives of, the study clearly described?

Partly

Are sufficient details of the methods provided to allow replication by others?

Yes

Are the datasets clearly presented in a useable and accessible format?

Not applicable

Reviewer Expertise:

tobacco control, health policy, statistics, epidemiology

I confirm that I have read this submission and believe that I have an appropriate level of expertise to confirm that it is of an acceptable scientific standard, however I have significant reservations, as outlined above.

# Response

**Published:** 2022-02-23

**Authors:** Masuma Pervin Mishu

**Affiliations:** University of York, UK

We would like to thank the peer reviewer for the valuable comments.

 Comment:

 Thus, it is important to prevent the initiation of ST in adolescents, which would protect against the health risks of ST use in adult life." This statement is not quite strong as authors only wrote about the ST prevalence which are 4.5% - 9.0% and added a reference about early smoking in adolescence. Perhaps authors can incorporate the prevalence over years in order to make this statement stronger and ST is a very important issue.

 Response:

 Thanks for your suggestion.

 No GYTS had been conducted in Bangladesh and Pakistan after 2013. Hence we could not report the prevalence over time as the data before 2013 will be too old. We have therefore not made any changes to this section.

 However, we added the following sentence to make the statement strong,

 ‘Evidence showed that most smokers start smoking in adolescence (NCBI 2012) and between one-third and one-half of adolescents who experiment with smoking become regular smokers (ASH 2018). The prevalence of ST use in adult population is high in these countries that poses serious disease burden (Siddiqi 2020). Since most adult tobacco users start ST use in adolescence, Since most adult tobacco users start ST use in adolescence, young people are targeted by the tobacco industry ( Wen et al., 2005) and ST manufacturers ( Connolly, 1995; Tobacco Free Initiative & WHO, 2002). Thus, it is important to to address tobacco use and prevent the initiation of ST in adolescents, which would protect against the health risks of ST use in adult life.’

 Comment:

 I am not really familiar with the educational system/stage in the study area. Is it similar in those three countries? Please add more detail about this.

 Response:

 Now we have added that-

 ‘The educational system is almost similar in these three countries. The age group of students in class 6-8 is almost similar in these three countries. ‘

 Comment:

1. For the FGD, please be more specific. How many participants per FGD?

Response:

 We have now added number of participants per FGD

 Comment:

 In order to increase the validity (I may say the honest response) authors have clarified that schoolteachers were not involved in questionnaire. However, I think there is a missing important point here. Were teachers also in the class or around students during the data collection? Teacher presence may give less comfort ability for students to fill in the questionnaire and this leads to weak validity. Authors may consider this.

 Response:

 We have now added the following sentence under ‘Risk, burdens and benefits’ to make the point clear-

 ‘Teachers were not involved in the process of quantitative data collection from students and were not present in the classroom during data collection keeping in mind that their presence might develop insecurity among students to answer honestly to fill in the questionnaire.’

 Comment:​​​​​​​ I would prefer to mention which version of STATA that was used.

 Response: We mentioned that STATA 16 will be used

 Comment:​​​​​​​ Longitudinal study is a quite long study and this study has mentioned that the follow up will be undertaken the next year (one year). I wonder if students move to another school due to higher educational stage, will there be any follow ups or will they be declared missing? The investigators need to consider this.

 Response: Due to practical considerations, students who move to another school due to higher educational stage will not be followed up. Longitudinal analysis techniques can accommodate varying follow-up points per participant.

 This point is now mentioned in the data analysis section. 

 Comment: ​​​​​​​

1. Although this is a feasibility study, I wonder whether the instrument for the quantitative study has been piloted or not. If it was not piloted, I may say the study outcomes are incomplete. Translating questionnaire into a local language especially for a study among adolescents is a bit challenging. Authors need to add more detail about this (e.g., piloting the translation, third party who translated, etc). I think this should be included as the study outcome. For example, 1) during the survey how many students were asking specific questions because some words or sentences from the local language in the questionnaire that they might not understand, and 2) feedbacks or comments from students about each specific questions during the FGD.

Response: Many thanks for your suggestion-

 We have now added the following sentences in the Methods,

 ‘A questionnaire, to be self-administered, for the students was developed and translated into the local language and checked by a native speaker. A pilot study to check all the arrangements before moving onto the feasibility study was conducted with at least 8-10 students in one school in each of the three counties two weeks prior to the baseline data collection for the feasibility study. We will revise the data collection tools in the light of feedback from the baseline data collection before carrying out follow-up data collection one year after the baseline data collection. We will revise the follow-up questionnaire if needed, based on the responses during the survey on how many students were making queries on some words or sentences from the local language in the questionnaire that they might not understand, and feedback or comments from students about each specific question.’

# Peer review: Reviewer response for version 1

**DOI:** 10.5256/f1000research.28468.r77408

**Published:** 2021-02-18

**Authors:** Praveen Jodalli

**Affiliations:** 1 Department of Public Health Dentistry, Yenepoya Dental College, Mangalore, Karnataka, India

**Title **

1. Has to be modified and can be more specific

**Abstract study**

1. objectives: objective 3 can be modified

**Methods and analysis:**

1. Mention whether the self administered questionnaire is for pilot study or longitudinal study.

**Keywords**

1. Feasibility can be replaced

**Introduction**

1. Use latest GATS statistics for India.
2. 5-7 are secondary school in India or primary school? Justify.
3. Second last paragraph mentioned that questionnaire will cover both cigarette and ST, but Title says only about ST.

**Study aim and objectives**

1. Modify third objective.
2. Change “assess” to evaluate for third objective.

**Methods**

1. Study has already started?
2. Mention the time period for the pilot study?

**Sampling strategy**

1. Last sentence: “Those schools provided written informed consent”-Who provided informed consent?
2. Consent from students?

**Selecting the sample and recruitment**

1. Two study groups?
2. Study groups doesn’t match with the title of  the article.
3. Criteria for selecting 25 students?
4. Figure 1 is incomplete. Total sample size, information on informed consent etc are missing ( mention n=?)
5. Figure 1- in text it is mentioned that participants are already recruited. But in figure it is mentioned that “will finally recruited”.
6. Number of investigators are missing.
7. What is parent/carer consent? For children under what age group?
8. Written assent or oral assent?(mention age group also).
9. Students were randomly selected- Method of randomization?
10. Why teachers are included in the study?
11. Eligibility criteria for teachers?

**Inclusion and exclusion criteria(Table 1)**

1. Mention 6-8 age group or class?
2. Mention about written or oral assessment?
3. Reason for exclusion of criteria 1,2,3

**Table 2**

1. Study outcomes- Why head teacher and class teachers.

**Outcomes to be measured**

1. Repetition of Table 2

**Collection of data from the students**

1. Why school teachers are included under this heading?

**Qualitative data**

1. Follow up period is not mentioned.
2. Verbal assent is enough for this age group?
3. What are the procedures for FGD?
4. How to assess the impact? Will you use same questionnaire for follow up?

**Collection of data from head teacher or other school representative and class teachers**

1. Why they are included in the study where the study population is students?
2. Will this data be included in the analysis?

**Data analysis**

1. How will you get the information on ST uptake and potential exposure?
2. How to evaluate the data?
3. How to compensate the attrition of participants?

**Method**

1. CONSORT reporting for pilot/feasibility trials?

**Consent**

1. Mention withdraw criteria.
2. What are the potential risk and burdens to the participants?

**Confidentiality**

1. How to maintain the confidentiality of the participants?
2. All eligible participants are in one class rooms, isn’t a breach of confidentiality?

**Data protection **

1. Why is identifiable information collected on the consent form?
2. Which method is used for data collection- questionnaire or interview?

Is the study design appropriate for the research question?

Yes

Is the rationale for, and objectives of, the study clearly described?

Partly

Are sufficient details of the methods provided to allow replication by others?

Yes

Are the datasets clearly presented in a useable and accessible format?

Partly

Reviewer Expertise:

Tobacco control, surveillance, public health, dental public health, clinical dentistry

I confirm that I have read this submission and believe that I have an appropriate level of expertise to confirm that it is of an acceptable scientific standard, however I have significant reservations, as outlined above.

# Response

**Published:** 2022-02-23

**Authors:** Masuma Pervin Mishu

**Affiliations:** University of York, UK

We would like to thank the peer reviewer for the valuable comments. The response of each comments are mentioned in the following Table:

 Reviewer 1

 Comment

 Response

**Title**

1. Has to be modified and can be more specific

The title has been changed to ‘Protocol for a feasibility study of longitudinal surveys to assess the impact of policies on tobacco use among school-going adolescents in South Asia’.

**Abstract study**

1. objectives: objective 3 can be modified

The third objective has changed to

 ‘To evaluate if the questionnaire can assess tobacco uptake and use, and their potential predictors.’

**Methods and analysis:**

1. Mention whether the self administered questionnaire is for pilot study or longitudinal study.

The question was changed to clarify that point, ‘At each time point, data on tobacco uptake and potential predictors will be collected from students via self-administered questionnaires that were designed for the longitudinal study.’

**Keywords**

1. Feasibility can be replaced

Feasibility was be replaced Feasibility study

**Introduction**

1. Use latest GATS statistics for India.
2. 5-7 are secondary school in India or primary school? Justify.
3. Second last paragraph mentioned that questionnaire will cover both cigarette and ST, but Title says only about ST.

 

1. Thanks for the suggestion. We have used the latest GATS statistics for India, GYTS 4 (2019). Reference: https://ntcp.nhp.gov.in/assets/document/National_Fact_Sheet_of_fourth_round_of_Global_Youth_Tobacco_Survey_GYTS-4.pdf
2. Secondary education in India is from 14 to 18 years as per reference. Elementary level education is till 14 years which is divided into two: 1. Primary education (Grade 1 to 5) and Upper primary level (Grade 6 to 8)

Reference:

https://niti.gov.in/planningcommission.gov.in/docs/plans/planrel/fiveyr/10th/volume2/v2_ch2_3.pdf

https://niti.gov.in/planningcommission.gov.in/docs/plans/planrel/fiveyr/10th/volume2/v2_ch2_2.pdf

1. We have now revised the term as school going adolescents throughout the manuscript to be consistent in reporting across three countries rather than saying secondary school. Now the title has been revised to tobacco use that captures both smoking and smokeless form of tobacco.

**Study aim and objectives**

1. Modify third objective.
2. Change “assess” to evaluate for third objective.

We have modified the third objective as suggested:

 ‘To evaluate if the questionnaire can assess tobacco uptake and use, and their potential predictors.’

**Methods**

1. Study has already started?
2. Mention the time period for the pilot study?

response:

1. The study was already started at the time when the manuscript was written. We mentioned that in the Methods and in the third sentence of the first paragraph of Methods:

‘In this section the processes that have already been conducted are described in past tense, and those still to do are described in the future tense.‘

 2. We mentioned the time period for the feasibility study, ‘The schools and students have been recruited and baseline data were collected between October 2019 and February 2020.’

 We mentioned that the follow-up data collection will be done one year after the baseline data collection

 We have now added the time period for the pilot study:

 ‘The pilot study to check all the arrangements before moving onto the feasibility study was conducted with at least 8-10 students in one school in each of the three counties two weeks prior to the baseline data collection’.

**Sampling strategy**

1. Last sentence: “Those schools provided written informed consent”-Who provided informed consent?
2. Consent from students? Response: We have now added the following sentence to make it clear Who provided informed consent?

‘The principal or head teacher of the school provided the written informed consent to participate in the study on behalf of the school.’

 Consent form were signed by the parents of the students then assent forms were signed from the students to participate in the study. The information on student assent was included in the manuscript.

**Selecting the sample and recruitment**

1. Two study groups?
2. Study groups doesn’t match with the title of the article.
3. Criteria for selecting 25 students?
4. Figure 1 is incomplete. Total sample size, information on informed consent etc are missing ( mention n=?)
5. Figure 1- in text it is mentioned that participants are already recruited. But in figure it is mentioned that “will finally recruited”.
6. Number of investigators are missing.
7. What is parent/carer consent? For children under what age group?
8. Written assent or oral assent?(mention age group also).
9. Students were randomly selected- Method of randomization?
10. Why teachers are included in the study?
11. Eligibility criteria for teachers?

Response:

1. The study was planned to be conducted among two groups of study participants: school students and schoolteachers which included head teacher or a representative of the school and class teachers.
2. In this feasibility study we wanted to test the feasibility of conducting the longitudinal study from the context of the students but also ensure we took account of views of the schoolteachers to inform the main study. However, the overall aim of the planned future longitudinal study is to ‘assess the impact of tobacco control policies on tobacco use among school going adolescents in South Asia’ as mentioned in the title
3. The inclusion criteria for selecting the students are mentioned in Table 1. However, as a feasibility study we did not conduct formal sample size calculations. The minimum number of 25 students per class was selected based on the discussion with the study partners from each country.
4. Figure 1 is an illustration of the process and stages of recruitment that we used. We did not present the actual numbers (n) here as these were not known at the time of writing the protocol (data compilation of three countries were under process).
5. We have changed the tense as past tense in Figure 1.
6. We have now added the number of Investigators in the first paragraph of the Methods section- ‘On average the number of investigators was 10-15 per country who conducted the data collection.’
7. We mentioned that ‘All students participating in this study were under 16 years old and therefore parental/carer consent was required for them to take part.’
8. We have added the age and made it clear that it was written assent.‘Students under the age of 16 years were asked to sign the assent form if they were willing to participate in the study.’
9. For the FGD we made the point clear-‘6-8 students per class were randomly selected by their class teachers to take part in the FGD, having previously secured parental consent and written assent from the students.‘
10. Teachers were involved in conducting the study in schools in terms of the process of selection, recruitment and collecting consent and assent forms from parents and students. It was important therefore to involve teachers in our research so that we could understand and be able to take into account any feedback they had when conducting the future longitudinal study.
11. We mentioned the eligibility criteria for teachers –‘schoolteachers which included headteacher or a representative of the school and class teachers of the classes participating in the study’

**Inclusion and exclusion criteria(Table 1)**

1. Mention 6-8 age group or class?
2. Mention about written or oral assent?
3. Reason for exclusion of criteria 1,2,3

Response:

1. Now we mentioned that ‘Students of 6th, 7th and 8th classes from the selected

schools, who have the ability to give written assent.

 2. Addressed in previous response 

 3. As we wanted to get the data on tobacco use from the general students and not from the students of special needs, therefore, we set the exclusion criteria 1,2,3 

**Table 2**

1. Study outcomes- Why head teacher and class teachers. Response: Please see above. (Response of point 10 of ‘Selecting the sample and recruitment’)

**Outcomes to be measured**

1. Repetition of Table 2

Response:

 We presented Table 2 to show the objectives in parallel to the study outcomes to make it easy to read and in the text we explained the points in detail.

**Collection of data from the students**

1. Why school teachers are included under this heading?

Under the point ‘Data collection methods’ we have two subheadings:

***1. Collection of data from the students***

***2. Collection of data from the headteacher or other school representative and class teachers***

 Response:

 School teachers are included under the second subheading. To make the point clear, we have now added numbers in the subheadings.

**Qualitative data**

1. Follow up period is not mentioned.
2. Verbal assent is enough for this age group?
3. What are the procedures for FGD?
4. How to assess the impact? Will you use same questionnaire for follow up?

Response:

1. We have now mentioned the follow up time
2. We already mentioned before-

‘6-8 students per class were randomly selected by their class teachers to take part in the FGD, having previously secured parental consent that included the FGD and  written assent from the students. In addition, verbal assent was obtained from the selected students again before the FGD commenced.’

 To avoid the repetition, we have now deleted the sentence related to assent from this paragraph.

 3. We briefly described the  procedure of FGD:‘6-8 students per class took part in the FGD. Topic guides were used to ensure consistency of discussion across schools, although the format was flexible to allow the students to raise additional issues they considered important. The discussions were conducted at the school in a private room by a field investigator and were digitally audio-recorded.’

 4. As mentioned,  in baseline FGD we will assess, ‘their views and experiences of being informed about the study, discussing the study with their parents, providing assent, and completing the questionnaire’

**We aimed to ‘assess the impact of tobacco control policies….’ in our main future longitudinal study not in this feasibility study.**

 A different topic guide will be used in the follow up FGD. We mentioned that-

  ‘The FGDs during the follow-up data collection will be focused mainly on students’ own and others’ tobacco uptake and use, and influences on this to assess the relevance and appropriateness of the questions in the follow-up questionnaire.’

**Collection of data from head teacher or other school representative and class teachers**

1. Why they are included in the study where the study population is students?
2. Will this data be included in the analysis?

Response:

 Please see above (Response of point 10 of ‘Selecting the sample and recruitment’)

 Yes, the qualitative data from the teachers will be used in qualitative analysis to assess the feasibility of conducting the longitudinal study in school setting from the perspective of the teachers.

**Data analysis**

1. How will you get the information on ST uptake and potential exposure?
2. How to evaluate the data?
3. How to compensate the attrition of participants?

Response:

1. We have now added that-

‘The questionnaire used for the survey incorporates questions related to tobacco uptake and potential exposure.’ The questionnaire will be available upon request to the corresponding author.

 2. We have now added that- ‘We will triangulate the findings obtained from the quantitative data related to the feasibility assessment and the qualitative study findings to evaluate the overall feasibility of conducting the future longitudinal study.’

 3. The feasibility study was set up to assess the attrition rates, not to compensate for them

**Method**

1. CONSORT reporting for pilot/feasibility trials?

Response:

 As it was not a pilot/ feasibility trial, so CONSORT reporting was not reported

**Consent**

1. Mention withdraw criteria.
2. What are the potential risk and burdens to the participants?

Response:

1. We have now added the withdraw criteria -

‘It was mentioned in consent and assent form that participants were free to withdraw any time as per their wish without showing any reason. In that case, no more data would be collected. The information already collected will be kept secure and still used in the analysis unless the participant specifically asks for the information to be removed.’

 2. We have added-‘Participants may have concerns about the risk of disclosure of their tobacco consumption practices and/or any breaches of confidentiality concerning their data or information provided. ‘

**Confidentiality**

1. How to maintain the confidentiality of the participants?
2. All eligible participants are in one class rooms, isn’t a breach of confidentiality?

Response:

1. We mentioned that- ‘Participation in this study was anonymous so any name or any identifiable details would not be disclosed. The questionnaires were identifiable and were coded with a study enrolment number. The participants were assured that no names would be associated with the data, which would be kept in a locked secured facility.’
2. We have added that-

‘The data collection was monitored by the investigators to ensure that no participant can view another participant’s responses in the questionnaire to maintain confidentiality’.

**Data protection**

1. Why is identifiable information collected on the consent form?
2. Which method is used for data collection- questionnaire or interview?

Response:

1. To make the point clear, we have now added- ‘Appropriate data protection and security procedures are put in place. Identifiable information was collected on the consent form in order to be able to match the students in the follow-up data collection.  Identifiable information collected on the consent form and codes were stored separately from the questionnaires (used for the quantitative data collection). Interview and FGD (related to the qualitative data collection) data were entered using the IDs allocated to the schools and student participants and also kept separate from the codes.’
2. The questionnaire was used for collection of quantitative data whereas In-depth interviews and focus group discussions were used for collection of qualitative data to explore feasibility aspects of the study.
